# Impact of a delayed diagnosis of vulvar cancer and its association with HIV infection: A 4-year review at a tertiary hospital in KwaZulu-Natal, South Africa

**DOI:** 10.4102/sajhivmed.v22i1.1272

**Published:** 2021-09-08

**Authors:** Ramakhosana S. Hlapane, Thandekile L. Khumalo, Bongumusa S. Makhathini, Jagidesa Moodley

**Affiliations:** 1 Department of Obstetrics and Gynaecology, Edendale Regional Hospital, Pietermaritzburg, South Africa; 2 Department of Obstetrics and Gynaecology, Faculty of Health Sciences, University of KwaZulu-Natal, Durban, South Africa; 3 Department of Obstetrics and Gynaecology, Grey’s Hospital, Pietermaritzburg, South Africa; 4 Women’s Health and HIV Research Group, Faculty of Health Sciences, Nelson R Mandela School of Medicine, Durban, South Africa

**Keywords:** vulvar cancer, HIV infection, young women HIV/HPV co-infection, HPV infection, HPV related cancers

## Abstract

**Background:**

Vulvar cancer is becoming more common in young women owing to the increased prevalence of co-infection with human papillomavirus and HIV.

**Objectives:**

The aim of this study was to determine the impact of the time interval from the diagnosis of vulvar cancer at the referring institution to the tertiary hospital and to evaluate the impact of HIV infection in the study population.

**Method:**

This was a retrospective descriptive chart review.

**Results:**

A total of 86 cases of vulvar cancer were analysed. The mean age was 48.2 ± 12.5. Sixty (69.8%) patients were under 50 years of age and eight (9.3%) under 30 years. The interval from the onset of symptoms to the diagnosis of cancer was > 12 months in 63 (73.3%) patients. Eighty-one (94.8%) had had symptoms treated multiple times prior to diagnosis. Seventy (81.4%) were referred to the tertiary institution within 3 months of the diagnosis of cancer. Seventy (81.4%) had concomitant HIV infection. Of those with CD4 counts of > 200 cells/mm^3^, 61.7% had early-stage vulvar cancer, while 38.3% had late-stage disease (*P* = 0.048). There was no association between the viral load and the Federation of Gynaecology and Obstetrics stage (*P* = 0.401). The primary treatment was surgery in 50%.

**Conclusion:**

Although the study was retrospective, we found that vulvar cancer was prevalent in younger patients with HIV infection. Higher CD4 counts were associated with early-stage disease. Early sampling of suspicious lesions can ensure early diagnosis of vulvar cancer and the initiation of therapeutic interventions, particularly in HIV-infected patients.

## Introduction

Vulvar cancer comprises 4% – 5% of female genital tract cancers.^1^ There are two types of vulvar cancer: keratinising squamous cell carcinoma, which usually occurs in post-menopausal women and is associated with lichen sclerosis or differentiated vulvar intraepithelial neoplasia, and basaloid squamous cell carcinoma, which commonly occurs in premenopausal women and is associated with high-risk subtypes of human papillomavirus (HPV) infection.^2^ Although vulvar cancer was traditionally thought to be common in the elderly, it is now reported to be occurring more commonly in young women because of the high prevalence of co-infection with HIV and HPV.^3^ According to Brinton et al., the mean age of presentation of vulvar cancer is over 70 years.^4^ However, about 10% of vulvar cancers are diagnosed at an age less than 50 years.^5^ A South African study by Butt et al. found a rising prevalence of vulvar cancer in young women, with the diagnosis being made at ages 10–15 years younger than those in high-income countries.^6^ Vulvar intraepithelial neoplasia is increasing worldwide owing to the rising incidence of HPV/HIV co-infection, especially in women during the reproductive phase of life.^7,8^

The probable causes of this delayed diagnosis have been noted to be multifactorial, involving both the patients and treating physicians. Patients may delay their presentation for vulvar symptoms for 2–16 months.^9^

Furthermore, histological sampling may be delayed for up to 12 months, as physicians primarily opt for medical treatment prior to sampling.^10^ This is because of hesitancy to biopsy the vulvar lesions or uncertainty about the best site to biopsy.^10^

There are currently no screening tests available for vulvar cancer. However, expert opinion recommends annual self-visual inspection of the external genitals by all women.^11^ Most patients diagnosed with vulvar cancer tend to present in the late stages of the disease. This has negative implications with respect to morbidity and poses financial challenges to the patient and a burden on the health system. The International Federation of Gynaecology and Obstetrics (FIGO) committee of Gynecologic Oncology descriptions date back to 1969 and were revised in 2009, and the revised staging applies to most malignancies of the vulva except melanoma.^11,12^

Most squamous vulvar malignancies are unifocal and involve the labia majora. However, vulvar cancer may be multifocal and involve sites such as the labia minora, clitoris and other parts of the perineum.^11^ It is therefore important to inspect the entire external genitalia for multifocal lesions.^11^ Any patient presenting with suspicious symptoms and signs must have a thorough evaluation and early histological sampling of lesions in order to avert delay in therapeutic interventions. There are no specific gross diagnostic features of vulvar cancer, and the only gold-standard diagnostic tool remains histological sampling of suspected vulvar lesions.^1^

In our analysis, we intended to determine the impact of the time interval between the onset of vulvar symptoms and the diagnosis of cancer, as well as the time interval from diagnosis at the base hospital to the referral tertiary institution in relation to the FIGO stage of the disease. We also sought to determine the association of HIV co-infection and stage of vulvar cancer, the characteristics of the patient demographics and the presenting symptoms at a tertiary facility.

## Methods

This was a retrospective study of all women with a histological diagnosis of vulvar cancer who were referred to the Gynae-Oncology Unit of a tertiary hospital in KwaZulu-Natal over a period of 4 years.

**FIGURE 1:**
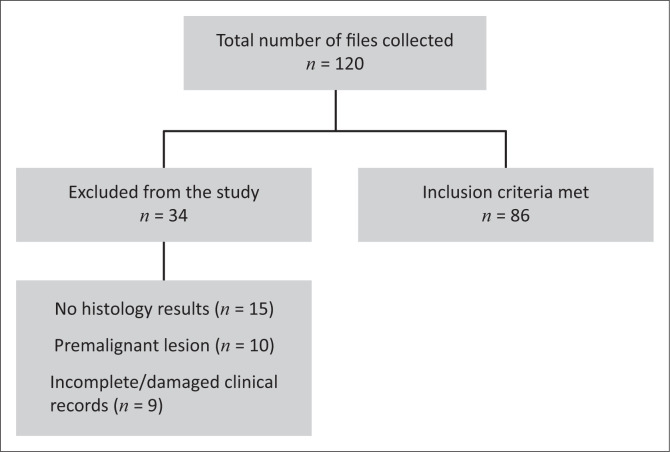
Flow diagram of the study population.

The gynaecology outpatient department register and records were used to identify the details of patients’ clinical records, which were then used to retrieve files from the medical registry. The relevant patient details were used to extrapolate the CD4 and viral load results from the National Health Laboratory Services website. A data abstraction tool was used to capture the required data from the clinical records that met the inclusion criteria during the study period. Clinical records with inadequate or incomplete data were excluded from the study (Figure 1).

The data were entered into a Microsoft Excel spreadsheet and exported to Statistical Package for Social Sciences (SPSS) version 25 software for statistical analysis. The descriptive statistics summary measures included proportions and percentages, means with standard deviations and medians with ranges. Proportions were compared using the chi-squared test or Fisher’s exact test. Continuous variable means were compared using unpaired *t*-tests for normally distributed data. A *P*-value of < 0.05 was regarded as indicating statistical significance.

## International Federation of Gynaecology and Obstetrics surgical stage

Early vulvar cancers are those where the disease is confined to the vulva with no lymph node involvement. Table 1 elaborates further the FIGO surgical stages of vulvar cancer.

**TABLE 1:** International Federation of Gynaecology and Obstetrics surgical staging of vulvar cancer.

Surgical stage	Description
**1**	Cancer limited to the vulva
1A	Lesion ≤ 2 cm in size, limited to the vulva or perineum and stromal invasion of ≤ 1.0 mm, but no lymph node metastasis
1B	Lesion > 2 cm in size or with stromal invasion of > 1.0 mm, confined to the vulva or perineum, with negative lymph nodes
**2**	Cancer of any size invading the adjacent perineal structures (the lower third of the urethra, lower third of the vagina and anus), with negative lymph nodes
**3**	Cancer of any size with/without invasion of the adjacent perineal structures, with positive inguino-femoral lymph nodes
3A	With 1 lymph node metastasis (≥ 5 mm), or With 1–2 lymph node metastases (< 5 mm)
3B	With 2 or more lymph node metastases (≥ 5 mm), or With 3 or more lymph node metastases (< 5 mm)
3C	With positive lymph nodes with extracapsular spread
4	Cancer extending to the other regional organs (upper two-thirds of the urethra, upper two-thirds of the vagina) or distant structures
4A	Cancer extending to: Upper urethral and/or vaginal mucosa, bladder mucosa, rectal mucosa, or fixed to the pelvic bone, or Fixed or ulcerated inguino-femoral lymph nodes
4B	Distant spread with pelvic lymph node involvement

### Ethical considerations

Ethical approval was obtained from the University of KwaZulu-Natal Biomedical Research Ethics Committee (BREC/00001294/2020). Provincial Health Authority approval was obtained from the National Health Research Database (KZ_202005_010), and institutional permission was provided by the senior management of the tertiary hospital. Clinical records were managed confidentially, and names or identification numbers were not used to identify the data abstraction tools.

## Results

### Demographics

A total of 120 files of women managed for vulvar cancer were collected and screened between 01 January 2015 and 31 December 2018 for inclusion in the study. Eighty-six files met the study inclusion criteria and were analysed. The majority of women in the study were of African ancestry and constituted 97.7% of the study group. The mean age of the women was 48.2 ± 12.5 years. The youngest woman was 23 years old, and 27 (31.4%) women were > 50 years.

### Clinical characteristics

The HIV status was known in 84 of the 86 cases (97.7%), with 70 (83.3%) of the 84 testing positive, 14 (16.7%) negative and two having an undocumented HIV status. The CD4 count was documented in 53 (75.7%) of those living with HIV. Forty-seven (89%) had a CD4 count of ≥ 200 cells/mm^3^, and only six (11%) had a CD4 count of ≤ 199 cells/mm^3^. The viral load was recorded in 30 (42.9%) of the HIV-infected patients, of whom 25 (36%) had viral suppression and five (7%) had an unsuppressed viral load. The viral load was not documented in 40 (57%) patient files. Sixty-nine (98.6%) of those living with HIV were on a first-line antiretroviral treatment (ART) regimen, and one (1.4%) was on second-line therapy. Table 2 further illustrates the demographic and clinical findings from this study.

**TABLE 2:** Demographics and clinical details of women with vulvar cancer (*n* = 86).

Characteristics	*N*	%	Mean ± s.d.
**Race**			
African ancestry	84	97.7	
Mixed race	1	0.01	
Caucasian	1	0.01	
**Age group (years)**			
< 30	8	9.3	
30–39	35	40.7	
40–49	16	18.6	
50–59	17	19.8	
> 60	10	11.6	
**Age**	-	-	48.2 ± 12.5
**Cervical cytology on referral**			
Done	36	41.9	
Not done	50	58.1	
**Cervical cytology result (*n* = 36)**			
Normal	8	22.0	
LGSIL	10	28.0	
HGSIL	18	50.0	
**HIV status (*n* = 86)**			
Positive	70	81.4	
Negative	14	16.3	
Unknown	2	2.3	
**ART (*n* = 70)**			
Regimen I	69	98.6	
Regimen II	1	1.4	
**CD4 count† (*n* = 70)**			
≥ 200	47	89.0	
≤ 199	6	11.0	
Missing data	17	17.0	
**Viral load (*n* = 70)**			
Suppressed	25	36.0	
Unsuppressed	5	7.0	
Missing data	40	57.0	

LGSIL, low-grade squamous intra-epithelial lesion; HGSIL, high-grade squamous intra-epithelial lesion; ART, antiretroviral treatment.

† , The low CD4 count below 200 cells/mm^3^ was based on Reference 13.

The majority of the women (30; 34.9%) presented with vulvar pain. Table 3 tabulates the patients’ signs and symptoms at presentation to a health establishment.

**TABLE 3:** Initial presenting symptoms, signs and treatment.

Characteristics	*n*	%
**Symptoms/signs**		
Vulvar pain only	29	34.9
Lump only	27	31.4
Ulceration only	5	5.8
Pruritus only	4	4.7
Vulvar pain + lump	15	17.4
Vulvar pain + ulceration	2	2.3
Vulvar pain + pruritus	2	2.3
Vulvar pain + lump + ulceration	1	1.2
**Management of initial symptom prior to diagnosis of vulvar cancer**		
Medical (simple oral analgesia and/or podophyllin)	82	82.0
Surgical excision or cauterisation of vulvar lesions	4	4.7
**Number of times symptoms treated prior to diagnosis of vulvar cancer**		
Once	5	5.8
≥ Twice	81	94.8

### Impact of HIV on International Federation of Gynaecology and Obstetrics stage

There was no statistically significant association between the level of the viral load and the initial FIGO stage (*P* = 0.401; 95% confidence interval [CI]: 0.393–0.453). However, there was a statistical significance between the CD4 count and FIGO stage (*P* = 0.048; 95% CI: 0.044–0.064). For patients with a CD4 count of ≤ 199/mm^3^, 50% had early-stage cancer, while 50% had late-stage cancer. However, at CD4 counts of > 200 cells/mm^3^, 61.7% had early-stage disease, while 38.3% had late-stage disease. The results are further demonstrated in Table 4.

**TABLE 4:** Association of HIV with International Federation of Gynaecology and Obstetrics stage.

Variables	FIGO stage at tertiary hospital								*P*	95% CI
	FIGO Stage 1		FIGO Stage 2		FIGO Stage 3		FIGO Stage 4			
	*n*	%	*n*	%	*n*	%	*n*	%		
**Viral load**										
Suppressed	6	7.0	7	8.1	9	10.5	3	3.5	0.401	0.393–0.453
Unsuppressed	1	1.2	1	1.2	3	3.5	0	0.0		
**CD4 count**										
≤ 199	1	1.2	2	2.3	3	3.5	0	0.0	0.048	0.044–0.064
≥ 200	13	15.1	16	18.6	15	17.4	3	3.5		

FIGO, International Federation of Gynaecology and Obstetrics; CI, confidence interval.

### Time of referral and International Federation of Gynaecology and Obstetrics stage at tertiary institution

The time interval between the onset of the symptoms and the diagnosis of vulvar cancer in the study group was delayed by 3–6 months in eight (9.3%) patients, 6–12 months in 15 (17.4%) patients and > 12 months in 63 (73.3%) patients.

Seventy (81.4%) patients first presented to the tertiary institution within 3 months from the referring facility. As demonstrated in Table 4, the disease was in Stage I in 25 (29.1%) women, Stage II in 24 (27.9%) women, Stage III in 25 (29.1%) women and Stage IV in 12 (14%) women.

### Treatment modalities and outcomes of vulvar cancer

The primary treatments were surgery (50%) and chemo-radiation (36%), followed by radical radiotherapy (14%), as described in Table 5.

**TABLE 5:** International Federation of Gynaecology and Obstetrics surgical stage and primary treatment at tertiary hospital.

Variables	*N*	%
**Referral time intervals (months)**		
< 3 months	70	81.4
3–6 months	11	12.8
6–12 months	5	5.8
> 12 months	0	-
**FIGO surgical stage**		
I	25	29.1
II	24	27.9
III	25	29.0
IV	12	14.0
**Primary treatment modality**		
Surgery	43	50.0
Chemo-radiation	31	36.0
Radical radiotherapy	12	14.0

*n* = 86.

FIGO, International Federation of Gynaecology and Obstetrics.

## Discussion

The main findings of our study were that most women presented at a young age, and most were living with HIV. The delays occurred with the sampling of suspicious lesions, which led to a delay in the diagnosis of vulvar cancer. Women with higher CD4 counts (≥ 200 cells/mm^3^) generally presented with early-stage vulvar cancer.

The study group comprised mainly women of African ancestry. This may be explained by the fact that the catchment areas of our tertiary hospital are predominantly populated by people of African ancestry.^14^ The mean age of women presenting with vulvar cancer at our tertiary hospital was 48.2 (± 12.5) years, and our study findings are comparable to a Nigerian study in which the mean age was found to be 49.7 years.^15^ A study from the United States evaluating demographics in young women with vulvar cancer found an even lower median age of 38 years.^16^ Other South African studies^6,17^ found the mean age to be 52.5 (± 15.5) years and 54.76 (± 16.59) years, respectively. The differences between our study population’s age and others may have been because of the high rates of concomitant HIV infection in our population. The available literature suggests that vulvar cancer is becoming more common in younger women because of the rising prevalence of HIV and HPV co-infection.^6^ Although there was minimal data on the status of HPV infection in our histology reports, we cautiously assumed that the high HIV prevalence was possibly associated with high rates of HPV co-infection.

The duration of patient-reported symptoms before diagnosis ranged from less than 3 months to greater than 12 months. In our study, we found that 73.3% of the patients had symptoms for more than 12 months before the diagnosis of vulvar cancer, with 43% presenting with advanced-stage disease category (FIGO Stages III and IV). Our findings differ from those of Lanneau et al., who found that 28.6% of patients had symptoms for more than 12 months prior to the diagnosis of vulvar cancer. Despite the prolonged symptoms, this study did not find a statistically significant correlation between the symptom duration and the clinical stage at presentation. In Lanneau’s study, only two of 56 (3.6%) women were HIV infected. Smoking, HPV exposure and other immunosuppressive comorbidities were identified as important predisposing factors for vulvar cancer in younger women.^16^ In a more recent local study with a larger sample size, the median duration of patient-reported symptoms before diagnosis was 6 months, and 53.3% of women had advanced-stage cancer FIGO (Stage III or higher).^6^ In this study, fewer women (23.7%) had HIV co-infection compared to the present study. Notably, HIV infection and its immunosuppression is associated with a high risk of metastasis of malignant lesions.^18^

In our analysis, most women had their symptoms treated medically (95.3%) and on multiple occasions (94.2%). The available literature recommends that symptoms such as persistent vulvar pruritus, vulvodynia, bleeding (unrelated to menstruation), skin discoloration, skin thickening or a lump, or an ulcer be treated as ‘red flags’ for vulvar cancer; such complaints warrant a detailed history, prompt assessment and investigation for vulvar cancer, especially in those who are immune compromised.^19^ Physician delay is usually because of a hesitancy to biopsy the vulva and resorting to offering medical treatment without a histological diagnosis.^10^

In our study, 83.3% women were HIV positive with access to healthcare facilities and could have benefited from early sampling and diagnosis of the vulvar cancer during their HIV treatment programme. In low- to middle-income countries, women tend to use alternative therapeutic modalities before seeking attention from a healthcare facility. In a local survey, about 70% of patients first sought help from traditional experts and only attended hospital when the symptoms became unbearable and the disease was advanced.^20^ Furthermore, women delay presentation, possibly because of a lack of recognition and interpretation of the seriousness of the symptoms of vulvar cancer and a hesitancy to report genital symptoms to a physician because of religious or cultural reasons. Although we could not extract data on patient-related factors in our study, there is a probability that these factors contributed to the delay of vulvar cancer diagnosis.

In our study, HIV infection was diagnosed in 83.3% of the women with vulvar cancer, compared to 23.7% and 25.0% found in other studies conducted in the Western Cape.^6,17^ The findings in our study suggest a higher HIV prevalence rate in our local setting. There is a high burden of HPV infection among young women in KwaZulu-Natal.^21^ It has been reported that HIV-positive women have a higher prevalence of HPV co-infection than their HIV-negative counterparts.^6,17,22^ This may be because of the effect of HIV immunosuppression, causing reduced rates of HPV clearance, which may cause high rates of persistent HPV infection or reactivation of latent HPV.^23^

In line with currently accepted HIV treatment guidelines, all HIV-infected women in our study were on ART. When we tested the association between HIV infection and FIGO stage in our study, we did not find a direct relationship between low viral load and FIGO clinical stage of vulvar cancer. At CD4 counts of ≥ 200 cells/mm^3^, 61.7% of women had early-stage cancer, while 38.3% had late-stage vulvar cancer. In our findings, patients with low CD4 counts had higher rates of advanced-stage disease; this finding needs to be treated with caution in view of the small sample size, and larger future multicentre studies may be needed in this regard. We did not come across literature that specifically demonstrated a relationship between the CD4 count and the progression of vulvar cancer; however, there is evidence that suggests that ART for HIV causes a modest 30.0% reduction of HPV clearance.^24^ Further studies are needed to determine the impact of CD4 count on disease progression.

## Interpretation

It must be noted that the findings from our study highlight the concerning lack of appropriate management of HIV-infected women, cervical cancer screening and management of suspicious vulvar lesions. Furthermore, the clinicians at district hospitals and HIV clinics where HIV-positive women are initially encountered need to be furnished with robust treatment algorithms for managing suspicious vulvar lesions in order to avoid delayed diagnosis of vulvar cancer. The Sexually Transmitted Infections Management Guidelines of South Africa currently recommends referral to a higher centre for care if perineal warts are inaccessible, numerous or measure > 10 mm.^25^ However, this guidance is limited in providing clear management of suspicious vulvar lesions at the primary healthcare level.

## Strengths and limitations

The retrospective study design, non-computed electronic records, small sample size and single institution data increase the risk of bias in our study. Both the clinical and statistically significant findings must be interpreted cautiously.

Furthermore, given the retrospective nature of the study and the fact that patients were referred from distant clinics, it was not easy to know the details of what medical or surgical treatment was given to patients when they initially presented with suspicious vulvar symptoms at their referring institutions.

## Recommendations and future research

We recommend that clinicians have a high index of suspicion of vulvar cancer, in particular when treating women presenting with vulvar symptoms and HIV co-infection, regardless of age. This should be followed by a prompt excisional biopsy of any abnormal vulvar lesions in order to aid early histological diagnosis. This package of care should form part of the district healthcare plans.

An avenue for future research would be to carry out a prospective multicentre study to determine the impact of the viral load and CD4 count on disease progression in terms of the FIGO stage and treatment outcomes in a larger sample size.

## Conclusion

Although our study is retrospective, we note that vulvar cancer is becoming prevalent in young women with underlying HIV infection. Early sampling of suspicious lesions and close follow-up is warranted to ensure the early diagnosis of vulvar cancer and the initiation of therapeutic interventions. Furthermore, the primary healthcare re-engineering model should intensify the training of clinicians in order to improve the clinical skills that may expedite a diagnosis of vulvar cancer. It is imperative that HIV clinicians strengthen health education on self-inspection of the vulva in their models of care.
